# Enhanced diagnosis of rabies and molecular evidence for the transboundary spread of the disease in Mozambique

**DOI:** 10.4102/jsava.v88i0.1397

**Published:** 2017-03-24

**Authors:** Andre Coetzer, Iolanda Anahory, Paula T. Dias, Claude T. Sabeta, Terence P. Scott, Wanda Markotter, Louis H. Nel

**Affiliations:** 1 Department of Microbiology and Plant Pathology, University of Pretoria, South Africa; 2 Central Veterinary Laboratory, Directorate of Animal Science, Mozambique; 3 Agricultural Research Council-Onderstepoort Veterinary Institute, Rabies Division, South Africa

## Abstract

Rabies is a neglected zoonotic disease with veterinary and public health significance, particularly in Africa and Asia. The current knowledge of the epidemiology of rabies in Mozambique is limited because of inadequate sample submission, constrained diagnostic capabilities and a lack of molecular epidemiological research. We wanted to consider the direct, rapid immunohistochemical test (DRIT) as an alternative to the direct fluorescent antibody (DFA) for rabies diagnosis at the diagnostic laboratory of the Central Veterinary Laboratory (CVL), Directorate of Animal Science, Maputo, Mozambique. Towards this aim, as a training exercise at the World Organisation for Animal Health (OIE) Rabies Reference Laboratory in South Africa, we performed the DRIT on 29 rabies samples from across Mozambique. With the use of the DRIT, we found 15 of the 29 samples (52%) to be negative. The DRIT-negative samples were retested by DFA at the OIE Rabies Reference Laboratory, as well as with an established real-time Polymerase chain reaction, confirming the DRIT-negative results. The DRIT-positive results (14/29) were retested with the DFA and subsequently amplified, sequenced and subjected to phylogenetic analyses, confirming the presence of rabies RNA. Molecular epidemiological analyses that included viruses from neighbouring countries suggested that rabies cycles within Mozambique might be implicated in multiple instances of cross-border transmission. In this regard, our study has provided new insights that should be helpful in informing the next steps required to better diagnose, control and hopefully eliminate rabies in Mozambique.

## Introduction

The aetiological agent of rabies, rabies virus (RABV), is a member of the *Lyssavirus* genus and accounts for tens of thousands of human deaths every year (World Health Organization [WHO] 2013). The number of animal deaths, particularly in reservoir species such as the domestic dog, far exceeds this number (Hampson et al. 2015; Nel 2013; WHO 2013). Rabies on the African continent is typically maintained within the mammalian order Carnivora, and the majority of deaths in resource-limited countries are associated with RABV cycles in dogs (*Canis lupus familiaris*) (WHO 2013). Several other carnivores such as the black-backed jackal (*Canis mesomelas*) and the bat-eared fox (*Otocyon megalotis*) are also able to maintain cycles of rabies and subsequently contribute to the spread of the disease (Bishop et al. 2010; Sabeta et al. 2007; Swanepoel 1993; Zulu, Sabeta & Nel 2009). According to anecdotal evidence, rabies has been present in North Africa for hundreds of years, but only became epizootic in sub-Saharan countries well into the 20th century (Nel & Markotter 2007; Nel & Rupprecht 2007). During the 1950s, rabies spread eastwards from the Mpumalanga province in South Africa to Mozambique, moving eastward to the Kingdom of Swaziland (1954) and then south-eastward to the KwaZulu-Natal (KZN) province of South Africa (1961 and 1974) (Swanepoel 1993).

The first introduction of rabies into the KZN province of South Africa probably occurred in 1961 when the disease spread from the Maputo district in southern Mozambique to the northern regions of the KZN province (Mansvelt 1962; Swanepoel 1993). The high density of the rural population in the coastal and midlands regions of KZN favoured the spread of rabies and the outbreak was only brought under control in 1968 (Mansvelt 1962; Swanepoel 1993). The second introduction of rabies into KZN occurred in 1974 when the disease once again crossed the border from Mozambique into the northern regions of the province. During this outbreak, rabies spread to the Eastern Cape province (1987), Ciskei area (now part of the Eastern Cape province) (1990s) and the Kingdom of Lesotho (1982) (Swanepoel 1993). It therefore appears that rabies cycles in Mozambique and South Africa may have overlapped since the 1960s. However, because of a lack of epidemiological surveillance throughout Mozambique the disease epidemiology is currently not well defined. This is primarily because of poor sample submission and limited diagnostic capabilities at the three existing veterinary laboratories, of which only the Central Veterinary Laboratory (CVL) in Maputo can perform the direct fluorescent antibody (DFA) test.

The classical method for rabies diagnosis is the DFA test (recognised as the gold standard test in 1973 by the World Health Organization, or WHO), which relies on detecting the presence of lyssavirus antigens by means of a fluorescein isothiocyanate-labelled antibody (Dean, Abelseth & Atanasiu 1996). Although the application of the DFA test in the hands of an experienced reader makes the test suitable for routine application, the proper application of the test in the developing world remains limited (Dürr et al. 2008; Weyer & Blumberg 2007). Because of the important role that post-mortem rabies diagnosis plays in disease management in animal populations (e.g. identifying disease outbreaks) as well as in risk assessment for the consideration of human prophylaxis, the development of more suitable diagnostic assays has taken precedence (Dürr et al. 2008; McElhinney, Fooks & Radford 2008; Wacharapluesadee & Hemachudha 2010).

Among the rabies diagnostic assays under development, the DRIT has shown promise in all applications to date and has proven to be particularly advantageous for low-resource settings (Coetzer et al. 2014b; Dürr et al. 2008; Lembo et al. 2006; Madhusudana et al. 2012; Saturday, King & Fuhrmann 2009). Previous studies relying on the implementation of the DRIT have indicated that it has a diagnostic efficacy equal to that of the DFA, but is easier to interpret, quicker to perform and requires a lower capital investment (Coetzer 2013). The routine application of the DRIT has been shown to be beneficial in low-resource countries such as Chad, Tanzania, Nigeria, Ethiopia, India, Iraq and Afghanistan (Adawa et al. 2014; Ali et al. 2014; Dürr et al. 2008; Lembo et al. 2006; Madhusudana et al. 2012; Saturday, King & Fuhrmann 2009). However, the relatively new assay needs to see widespread evaluation and implementation in order to gain credibility as a reliable routine diagnostic test that can then be considered for possible World Organisation for Animal Health (OIE) accreditation.

**FIGURE 1:**
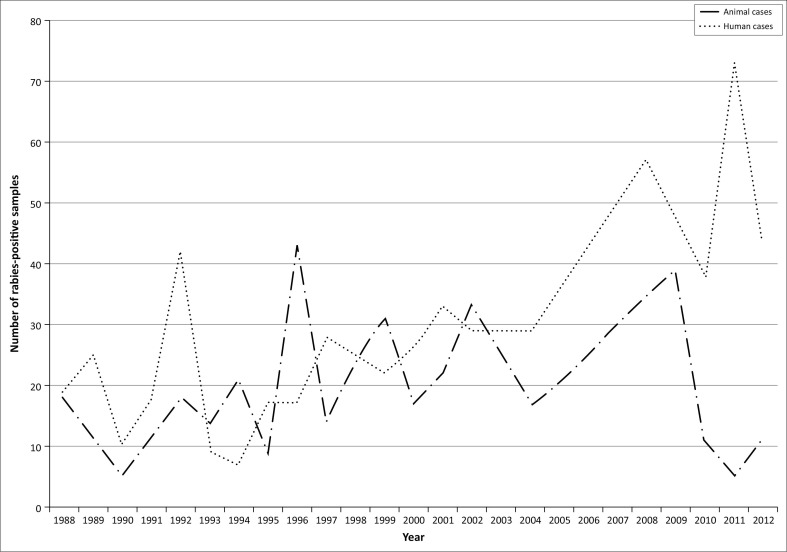
The number of animal and human samples diagnosed as rabies-positive at the Central Veterinary Laboratory in Maputo, Mozambique, over the last 25 years (1988–2012).

Over the past 25 years (1988–2012), rabies diagnostics in Mozambique have been inconsistent because of poor surveillance capacity, with specific reference to limited sample submissions and lack of diagnostic capabilities. As an illustration of the consequence of such limited surveillance, the number of human rabies cases (clinical diagnosis) for some years outnumbered confirmed rabies cases in the reservoir and vectors species, *viz.* dogs (southern and eastern African Rabies Group n.d.). Nevertheless, an average of 20 animal samples per annum were diagnosed as rabies-positive by means of the DFA test at the CVL in Maputo (Figure 1) (southern and eastern African Rabies Group n.d.). To date, the only molecular epidemiological study to include isolates from Mozambique was an investigation focused on the genetic relatedness of RABV isolates in the Mpumalanga province of South Africa. In that study, nucleotide sequences of two RABV isolates from Mozambique were compared to RABV sequences from Mpumalanga province and were delineated into a distinct cluster (Mkhize et al. 2010).

With a view to simplify and improve the rabies diagnostic capability at the CVL in Mozambique, we implemented the DRIT in a training exercise using a cohort of 29 samples previously confirmed as DFA-positive in Mozambique. We also sequenced the G-L RABV cDNA obtained from positive samples towards obtaining a molecular epidemiological view of the relationships among these viruses and with those viruses known from countries sharing borders with Mozambique. In this study, we aim to determine whether active cross-border spread of rabies between Mozambique and its neighbouring countries occurs, as this finding is important in structuring and evaluating regional plans for rabies control and elimination.

## Research method and design

### Sample cohort used in the study

This study was carried out at the OIE Rabies Reference Laboratory at the Agricultural Research Council-Onderstepoort Veterinary Institute (ARC-OVI), Rabies Division, South Africa. A total of 29 brain tissue samples (any available brain material) were subjected to routine DFA testing at the CVL in Maputo, Mozambique and all of the samples were diagnosed rabies-positive. The samples formed part of a repository of samples stored below -20 °C for an extended period of time (1993–2013) at the CVL (Table 1). The samples were then shipped to the South African laboratory for the DRIT training exercise. The conjugate used with the original DFA test and all subsequent antibody-based tests described here was the anti-ribonucleoprotein polyclonal antibody (PAb) preparation produced by the ARC-OVI (Perrin 1973).

**TABLE 1:** Neuronal tissue sample cohort from Mozambique depicting the initial diagnostic results from the Central Veterinary Laboratory in Maputo, Mozambique, the diagnostic discrepancies and their independent antigenic and molecular confirmation at the Agricultural Research Council-Onderstepoort Veterinary Institute in South Africa.

Sample ID	Animal	Year of isolation	Province of origin in Mozambique	DFA result (CVL in Mozambique)	DRIT result (CVL in South Africa	DFA result (CVL in South Africa)	PCR results (South Africa)
298/93	Canine	1993	Gaza	Positive	Positive	Positive	Positive
348/93*	Canine	1993	Maputo	Positive	Negative	Negative	Negative
675/96*	Canine	1996	Inhambane	Positive	Negative	Negative	Negative
1080/96*	Canine	1996	Maputo	Positive	Negative	Negative	Negative
603/98*	Canine	1998	Maputo	Positive	Negative	Negative	Negative
186/99	Canine	1999	Gaza	Positive	Positive	Positive	Positive
572/99	Canine	1999	Gaza	Positive	Positive	Positive	Positive
633/00	Canine	2000	Nampula	Positive	Positive	Positive	Positive
472/03*	Canine	2003	Maputo	Positive	Negative	Negative	Negative
174/04*	Canine	2004	Maputo	Positive	Negative	Negative	Negative
175/04*	Canine	2004	Maputo	Positive	Negative	Negative	Negative
315/04	Canine	2004	Maputo	Positive	Positive	Positive	Positive
44/05*	Canine	2005	Maputo	Positive	Negative	Negative	Negative
56/05*	Canine	2005	Maputo	Positive	Negative	Negative	Negative
74/05*	Canine	2005	Maputo	Positive	Negative	Negative	Negative
162/05*	Canine	2005	Maputo	Positive	Negative	Negative	Negative
232/05	Canine	2005	Nampula	Positive	Positive	Positive	Positive
378/05*	Canine	2005	Maputo	Positive	Negative	Negative	Negative
558/05	Canine	2005	Inhambane	Positive	Positive	Positive	Positive
659/05	Canine	2005	Sofala	Positive	Positive	Positive	Positive
687/05	Canine	2005	Gaza	Positive	Positive	Positive	Positive
131/12	Domestic cat	2012	Manica	Positive	Positive	Positive	Positive
482/12	Canine	2012	Maputo	Positive	Positive	Positive	Positive
586/12*	Canine	2012	Maputo	Positive	Negative	Negative	Negative
1018/12	Bovine	2012	Gaza	Positive	Positive	Positive	Positive
1174/12	Canine	2012	Nampula	Positive	Positive	Positive	Positive
196/13*	Canine	2013	Niassa	Positive	Negative	Negative	Negative
233/13	Canine	2013	Maputo	Positive	Positive	Positive	Positive
278/13*	Canine	2013	Sofala	Positive	Negative	Negative	Negative

* Diagnostic discrepancies: direct fluorescent antibody-positive samples from Mozambique diagnosed negative using the direct, rapid immunohistochemical test assay and subsequently confirmed negative by means of the direct fluorescent antibody assay at the Agricultural Research Council-Onderstepoort Veterinary Institute, Rabies Division, in South Africa.

DFA, direct fluorescent antibody; CVL, Central Veterinary Laboratory; DRIT, direct, rapid immunohistochemical test; PCR, polymerase chain reaction.

### Direct, rapid immunohistochemical test

All of the rabies samples (*n* = 29) were subjected to the direct, rapid immunohistochemical test (DRIT) diagnostic assay according to the standard operating procedure using a biotinylated PAb preparation (ARC-OVI, Rabies Division) (Coetzer et al. 2014b; Coetzer, Nel & Rupprecht 2014a). Both positive and negative controls were included in every run. The DRIT was performed blindly by two diagnostic technicians: one from the South African laboratory and the other from the Mozambican laboratory. Although both diagnosticians were familiar with the DFA, the diagnostician from Mozambique underwent training on the implementation and interpretation of the DRIT using this sample cohort. Thus, the samples in this study were the first DRIT-diagnosed samples that the diagnostician had interpreted. The South African diagnostician, who hosted the training, was thus the only individual who had extensive exposure to the DRIT (Coetzer et al. 2014b).

Briefly, a single touch impression was made from the brain tissues by placing a small amount of homogenised material on clean tissue paper. Touch impressions of the samples were allowed to air-dry for 5 min before being submerged in 10% neutral buffered formalin (Sigma-Aldrich, Johannesburg, South Africa) for 10 min. After fixation, the touch impressions were rehydrated by dip rinsing the slides in TPBS buffer (PBS, pH of 7.5 [Whitehead Scientific, Cape Town, South Africa] containing 1% Tween 80 [Merck, Johannesburg, South Africa]) and then submerged in 3% hydrogen peroxide (Merck) for 10 min at room temperature in order to halt all endogenous peroxidase activity. Subsequent to the hydrogen peroxide flooding, the slides were dip rinsed in fresh TPBS buffer and the excess buffer was shaken from the slides. The areas surrounding the smear impressions were blotted dry using fresh paper towel. Biotinylated PAb preparation (1:600 working concentration) was applied dropwise until the impression was completely covered. After the application of the antibody, the slides were placed in a humidity chamber, incubated at room temperature for 10 min and subsequently dip rinsed in fresh TPBS buffer. The excess buffer was shaken from the slides, and the areas surrounding the smear impressions were blotted dry using a fresh paper towel. All of the touch impressions were covered in a ready-to-use solution of 2 μg/mL streptavidin-peroxidase (Kirkegaard and Perry Laboratories, Gaithersburg, MD), after which the slides were transferred to a humidity chamber. The humidity chamber was incubated at room temperature for 10 min and the slides were dip rinsed in fresh TPBS buffer. The excess buffer was shaken from the slides and the areas surrounding the smear impressions were blotted dry using a fresh paper towel. The impressions were covered in a working solution of the 3-amino-9-ethylcarbazole (AEC) chromogen (Sigma-Aldrich), and the slides were transferred to a humidity chamber and incubated at room temperature for 5 min. After sufficient staining had occurred, the slides were submerged in distilled water. The touch impressions were counterstained with a 1:2 dilution of Gill’s formulation #2 (Sigma-Aldrich) for 2 min before they were dip rinsed in distilled water in order to wash away the residual counterstain. Finally, the slides were mounted with a water-soluble mounting medium (1xPBS [Whitehead Scientific]/glycerol [Sigma-Aldrich] prepared 1:1) and examined by light microscopy (Nikon, Alphashot YS) at both 200x and 400x magnification in order to score the respective immunoreactivity based on both the presence and staining intensity of the visible red inclusions present on the blue cellular background (Coetzer et al. 2014a).

### Direct fluorescent antibody test

The DFA diagnostic assay (CDC 2003; Dean et al. 1996) was repeated at the ARC-OVI, Rabies Division, on all of the samples (*n* = 29). One technician from the ARC-OVI Rabies Division performed the DFA test, and two experienced microscopists interpreted the results in a blind reading in order to eliminate bias.

### Viral RNA extractions, polymerase chain reaction and sequencing

The total RNA of all of the neuronal tissue samples (*n* = 29) was extracted using the TRI Reagent^®^ (Sigma-Aldrich) according to the manufacturer’s instructions. A reverse transcription polymerase chain reaction (PCR) was performed on the 14 confirmed rabies-positive samples using the G(+) and L(-) primers (Sacramento, Bourhy & Tordo 1991; Von Teichman et al. 1995), which amplifies the cytoplasmic domain of the glycoprotein gene and the adjacent G-L intergenic region of the RABV genome. Viral amplification was observed in all but one sample (1174/12). Because this sample (1174/12) was a low DFA-positive, the lack of amplification was likely because of advanced tissue decomposition. The PCR-positive products obtained from the remaining 13 samples were electrophoresed on a standard 1% agarose gel and subsequently gel-extracted and purified using the Wizard^®^ SV Gel and PCR Clean-Up System according to the manufacturer’s instructions (Promega, Madison, WI).

Both the forward and reverse strands of the purified PCR amplicons were sequenced using the PCR primers and the BigDye^®^ Terminator v3.1 Sequencing Reaction Kit according to the manufacturer’s instructions (Applied Biosystems, Foster City, CA) and sequenced using an ABI 3100 automated capillary sequencer situated at the University of Pretoria. With the use of the CLC Main Workbench software (CLC Bio, Version 7.0), the consensus sequences were trimmed to 592 nucleotides (nt), representing the cytoplasmic domain of the glycoprotein gene and the adjacent G-L intergenic region of the RABV genome (bases 4767–5358 according to the Challenge Virus Standard-11 rabies genome, GenBank accession number: GQ918139). The final sequences were subsequently submitted to the National Center for Biotechnology Information (NCBI) GenBank and allocated unique accession numbers (KM262037-KM262049) (Table 2).

**TABLE 2:** Panel of rabies viruses from Mozambique and neighbouring countries included in the phylogenetic analysis performed in this study.

Virus number	Reference number	Year of isolation	Species	Country	Province or region	GenBank accession number
1	RV2772	2010	*Canine*	Tanzania	Pwani	KF155002
2	722/86	1986	*Canine*	Malawi	Unknown	GQ983529
3	275/91	1991	*Canine*	Zambia	Unknown	GQ983449
4	d20896	1992	*Canine*	Zimbabwe	Masvingo	AF177062
5	d22547	1994	*Canine*	Zimbabwe	Mashonaland Central	AF177070
6	d24299	1999	*Canine*	Zimbabwe	Masvingo	AF177073
7	d85/04	2004	*Canine*	Swaziland	Hhohho	FJ842763
8	j45/94	1994	*Jackal*	South Africa	Limpopo	EF686064
9	d208/99	1999	*Canine*	South Africa	Limpopo	EF686061
10	d224/03	2003	*Canine*	South Africa	Limpopo	EF686111
11	d370/05	2005	*Canine*	South Africa	Limpopo	EF686082
12	d416/05	2005	*Canine*	South Africa	Limpopo	EF686098
13	d197/06	2006	*Canine*	South Africa	Limpopo	EF686152
14	572/09	2009	*Canine*	South Africa	Limpopo	GU808513
15	d536/96	1996	*Canine*	South Africa	Mpumalanga	EF686057
16	d1024/99	1999	*Canine*	South Africa	Mpumalanga	EF686068
17	d110/02	2002	*Canine*	South Africa	Mpumalanga	EF686086
18	d187/04	2004	*Canine*	South Africa	Mpumalanga	EF686102
19	d221/06	2006	*Canine*	South Africa	Mpumalanga	EF686146
20	d127/07	2007	*Canine*	South Africa	Mpumalanga	FJ842742
21	d180/07	2007	*Canine*	South Africa	Mpumalanga	FJ842743
22	d681/07	2007	*Canine*	South Africa	Mpumalanga	FJ842751
23	d1147/07	2007	*Canine*	South Africa	Mpumalanga	FJ842755
24	d1117/08	2008	*Canine*	South Africa	Mpumalanga	FJ842734
25	d1133/08	2008	*Canine*	South Africa	Mpumalanga	FJ842735
26	d755/95	1995	*Canine*	South Africa	KwaZulu-Natal	AF303081
27	KZNdg137/03	2003	*Canine*	South Africa	KwaZulu-Natal	DQ841438
28	KZNdg170/03	2003	*Canine*	South Africa	KwaZulu-Natal	DQ841442
29	KZNdg407/03	2003	*Canine*	South Africa	KwaZulu-Natal	DQ841504
30	d333/06	2006	*Canine*	Mozambique	Maputo	EU123929
31	g421/06	2006	*Caprine*	Mozambique	Cabo Delgado	EU123930
32	d520/06	2006	*Canine*	Mozambique	Nampula	EU123931
33	d529/06	2006	*Canine*	Mozambique	Manica	EU123932
34	d561/06	2006	*Canine*	Mozambique	Maputo	EU123933
35	d804/06	2006	*Canine*	Mozambique	Manica	EU123934
36	MOZdg298/93*	1993	*Canine*	Mozambique	Gaza	KM262037
37	MOZdg186/99*	1999	*Canine*	Mozambique	Gaza	KM262038
38	MOZdg572/99*	1999	*Canine*	Mozambique	Gaza	KM262039
39	MOZdg633/00*	2000	*Canine*	Mozambique	Nampula	KM262040
40	MOZdg315/04*	2004	*Canine*	Mozambique	Maputo	KM262041
41	MOZdg232/05*	2005	*Canine*	Mozambique	Nampula	KM262042
42	MOZdg558/05*	2005	*Canine*	Mozambique	Inhambane	KM262043
43	MOZdg659/05*	2005	*Canine*	Mozambique	Sofala	KM262044
44	MOZdg687/05*	2005	*Canine*	Mozambique	Gaza	KM262045
45	MOZfel131/12*	2012	*Domestic cat*	Mozambique	Manica	KM262046
46	MOZdg482/12*	2012	*Canine*	Mozambique	Maputo	KM262047
47	MOZbov1018/12*	2012	*Bovine*	Mozambique	Gaza	KM262048
48	MOZdg233/13*	2013	*Canine*	Mozambique	Maputo	KM262049
49	192J09 (Phylogenetic tree root)	2009	*Jackal*	Namibia	Kunene	JX473839

* Denotes the samples that formed part of the sample cohort included in this study.

All of the negative samples from the DFA at the ARC-OVI, Rabies Division were subjected to an established ‘one-step’ quantification real-time PCR assay (Coertse et al. 2010) targeting the RABV nucleoprotein. The decomposed DFA-positive sample (1174/12) was also included for confirmation (Table 1).

### Phylogenetic analysis

The phylogenetic analysis encompassed the sequences obtained from the Mozambique samples provided in this study as well as G-L intergenic region sequences available for selected African countries bordering Mozambique (eastern South Africa, Swaziland, Zimbabwe, Malawi, Zambia and Tanzania) (Table 2). The root of the phylogenetic tree is a comparative sequence that can then provide evidence of relatedness of sequences within a defined geographical area (Mozambique) and consists of a distantly related, but still relevant, sequence. Thus, a jackal isolate from Namibia (192J09) was used to root the tree.

An alignment of the collection of sequences was created using the ClustalW subroutine of the BioEdit software (Hall 1999) and a maximum likelihood phylogenetic analysis was performed using the PhyML software (Version 3.1) with an estimated bootstrap support of 1000 replicates and five possible starting trees. The best fitting DNA substitution model (TPM1+G model) was selected using the Akaike’s information criterion determined using the JModel software (Version 2.1.3).

## Results

### Rabies diagnosis using the direct, rapid immunohistochemical test

Fourteen of the 29 samples (48%) were found to be lyssavirus-positive using the DRIT. All of the samples from Mozambique were retested using the DFA at the ARC-OVI laboratory in South Africa in order to confirm the DRIT results. The results from the DFA in South Africa correlated with those of the DRIT (Table 1). These findings thus suggested that 52% of the samples from Mozambique were in fact false positive.

The diagnostic results, based on the direct detection of viral antigen (*n* = 14 rabies-positive by DRIT and DFA and *n* = 15 rabies-negative by DRIT and DFA), were subsequently confirmed by PCR (Table 1). All but one of the samples that were DRIT and DFA-positive were subsequently sequenced for the molecular phylogenetic analyses, whilst the DRIT-negative samples were tested using a real-time PCR for further confirmation of the negative result. The DFA-positive sample that could not be amplified by conventional PCR (1174/12) was confirmed low-positive using real-time PCR.

### Molecular epidemiology

**FIGURE 2:**
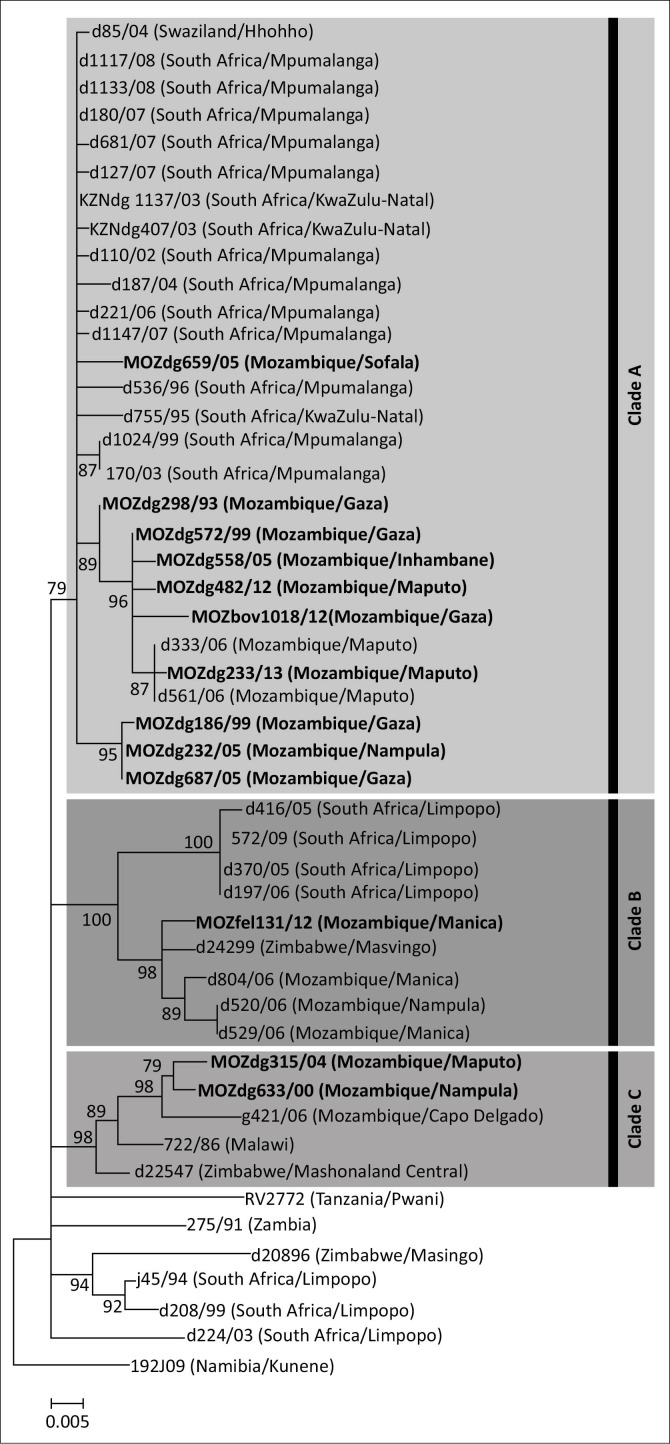
Maximum likelihood tree of 592 nucleotide (bases 4767–5358 according to the Challenge Virus Standard-11 rabies genome, GenBank accession number: GQ918139) sequences of the cytoplasmic domain of the G-L intergenic region for mammalian species (canine, feline, ovine, mongoose and domestic livestock) originating from selected sub-Saharan African countries. The horizontal branch lengths are proportional to the similarity of the sequences within and between groups and all branches with less than 75 bootstrap supports were collapsed. A Namibian jackal sequence (isolate 192J09) was used to root the tree.

**FIGURE 3:**
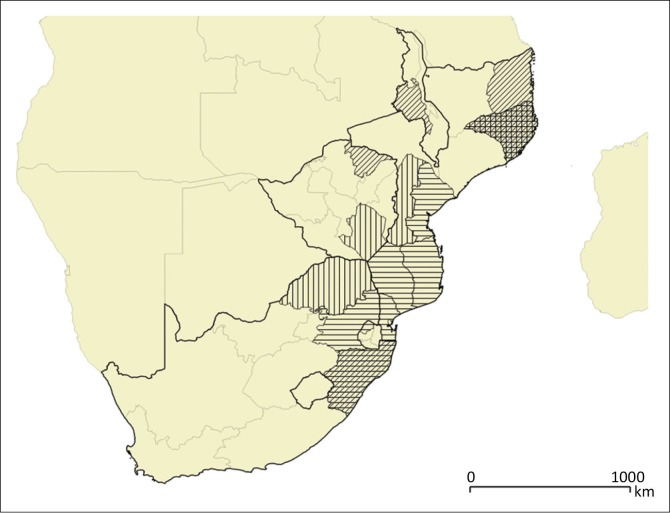
Illustrated map showing the inferred cross-border spread of endemic dog rabies between Mozambique and its neighbours. Only the provincial locations have been indicated to provide a broad overview of the samples’ locations. Samples forming part of Clade A have been indicated with horizontal lines, Clade B with diagonal lines and Clade C with vertical lines. The jackal sequence from Namibia (192J09) was not included in the map as it was only used as a root for the phylogenetic tree.

Phylogenetically, the collection of viral sequences was found to segregate into three main clades (Figure 2), each of which was subsequently found to correlate with specific geographical domains as identified by the province or region of virus origin (Figure 3).

The first clade, Clade A, consisted of RABV sequences obtained from RABV-infected dogs in Mozambique (Maputo, Gaza, Inhambane, Sofala and Nampula provinces), Swaziland and South Africa (KwaZulu-Natal and Mpumalanga provinces) (Figure 2). Our results suggest and confirm a trend of cross-border spread similar to that of an earlier study that investigated the re-emergence of dog rabies in the Mpumalanga province of South Africa (Mkhize et al. 2010). The branching clusters observed within Clade A illustrated that the viruses in this clade are closely related, suggesting that a single RABV strain historically circulated through Swaziland, Mozambique and eastern South Africa (Mkhize et al. 2010).

The second clade, Clade B, consisted of RABV sequences from RABV-infected dogs found in Mozambique (Manica and Nampula provinces), Zimbabwe (Masvingo province) and South Africa (Limpopo province) (Figure 2). Evidence of an endemic cycle in this region has previously been noted in another study where the cross-border spread of rabies between Zimbabwe and South Africa was suggested (Cohen et al. 2007). However, our study provides evidence that viruses found in Mozambique can also be linked to this cycle.

The third clade, Clade C, highlighted genetic relatedness between isolates originating from Mozambique (Cabo Delgado and Nampula provinces), Malawi and Zimbabwe (Mashonaland Central province) (Figure 2). To our knowledge, this is the first time that a close phylogenetic relationship between RABV from northern Mozambique and its neighbours to the west has been demonstrated.

## Discussion

We analysed samples that were found to be rabies-positive and were subsequently stored at -20 °C at the CVL in Maputo, Mozambique. After shipment to our laboratories in South Africa, 48% of these samples could be confirmed as rabies-positive with the use of DFA, DRIT and real-time PCR. There could be several reasons for the negative result in a fairly high percentage of the samples. Viral tropism could be one such reason, as only a portion of the brain material was shipped. However, all of the samples were homogenised prior to testing, which limits the likelihood of poor antigen distribution. A more likely possibility could be the degradation of antigens and genetic material to the point where these could not be detected by any of the methods we have applied. The maintenance of constant temperature storage of brain tissue samples subsequent to the application and interpretation of any diagnostic test is of the utmost importance (McElhinney et al. 2014). Because of limited infrastructure in Mozambique, power-spikes or power-outages at the CVL could indeed have resulted in the loss of cold temperature storage of the brain tissue samples. If the cold chain was not maintained during sample storage, the putrefaction of the brain tissue could explain the degradation of viral antigens and the subsequent lack of any positive signals in either the DRIT or DFA. On the contrary, these diagnostic discrepancies could reflect an initial diagnostic test that was poorly executed and in which non-specific fluorescence was misinterpreted to represent a positive diagnosis. Samples that have undergone tissue degradation and putrefaction (because of sub-optimal sample shipment conditions) could produce a large amount of non-specific fluorescence because of the presence of bacterial or fungal contamination (Mani & Madhusudana 2013). In the hands of an inexperienced reader, or a reader relying on an improperly calibrated fluorescent microscope, the non-specific fluorescence could be misinterpreted as positive signals, resulting in false positive results. In situations where microscopes are not functioning optimally, the addition of counter-stains such as Evans Blue should be considered to improve the contrast between the tissue and the fluorescent signal (CDC 2003).

Although the degradation of viral antigen during long-term storage is a very real issue in developing countries, a further factor should also be considered in these settings. The misinterpretation of non-specific fluorescence signals is a real danger in cases of poorly maintained fluorescent microscopes and other infrastructure. In such cases, the DRIT would be a superior test that is less sensitive to microscope issues. The sensitivity and specificity of the DRIT has been shown to be comparable with that of the DFA, yet an overarching benefit of the DRIT is the ease of differentiation between a positive and a negative result by trained personnel. This is because of the reduction in visible interfering background staining and the reduced reliance upon stringently calibrated equipment. Furthermore, archival samples are subject to degradation, resulting in a larger proportion of background fluorescence when using the DFA test (Aguiar et al. 2013; Lopes, Venditti & Queiroz 2010; Rojas Anaya et al. 2006). On the contrary, the DRIT does not rely on fluorescence and thus largely eliminates any potential misinterpretations of results because of the limited interfering background staining caused by degraded or archival samples. In addition, with the increasing availability of commercial reagents or antibodies and comparable running costs (unpublished data), the DRIT is a more practical test in countries where the training of personnel may not be ideal and resources are limited.

The routine collection and accurate diagnosis of brain samples provides the foundation for any rabies surveillance network as it permits the only true assessment of the disease burden. As shown in this study, the subsequent application of molecular epidemiology provides further resolution that can be used to apply active surveillance or strategic disease intervention campaigns. To our knowledge, the phylogenetic component of this study represents the first instance of the use of molecular epidemiology to investigate endemic rabies in Mozambique. The results were helpful in advancing our understanding of virus cycles and the transboundary movement of viruses in the larger region. Indeed, this study has shown that host species in Mozambique harbour several different rabies cycles, both geographically defined and shared with its immediate regional neighbours. We consider this finding supportive of anecdotal evidence of the transboundary spread of rabies in south-eastern Africa during the past century. The porous nature of national borders on the African continent has been demonstrated on more than one occasion (Mkhize et al. 2010), and transboundary transmission of rabies is not unexpected. Any control interventions, typically on a national level, would also need to consider the regional perspective in the long term. This regional approach will ensure that neighbouring countries work towards the same objectives by creating buffer zones against the reintroduction of the disease.

The Bill & Melinda Gates Foundation, in cooperation with the WHO, launched pilot canine rabies elimination programmes in three dog rabies endemic territories (South Africa, Tanzania and the Philippines) in 2009. The KZN province was selected as the South African study location because of the unique challenges the province faces for rabies control and elimination. KZN has achieved large-scale success since the programme was initiated, and reached a milestone in 2010 when the province celebrated 1 year without a diagnosed case of human rabies for the first time in 20 years (International Coordinating Group [ICG] 2014). The cross-border spread observed in this study indicates that a structured meta-population is most likely present. Canine populations on either side of the South African and Mozambican border are interconnected and are linked by migration, interaction and subsequent disease transmission as evidenced in Clade A (Figures 2 and 3). In a study investigating the use of oral vaccines on raccoon meta-populations separated by natural barriers, it was found that the construction of disease corridors on the far side of the natural barriers surrounding disease control regions was essential for sustained control (Russel, Real & Smith 2006). Therefore, despite the successes observed in KZN, the regional control of rabies will never be sustainable unless control strategies are implemented in neighbouring countries. Although vaccinations are currently undertaken in Mozambique, there is no structured disease control programme and vaccinations are performed sporadically only when vaccines are available (Rodrigues 2011). Low or sporadic vaccination coverage of a target population may have a detrimental effect and can possibly correlate with the persistence of rabies (Townsend et al. 2013; WHO 2013). Therefore, a carefully planned and coordinated rabies control programme, which is expanded in a wave-like manner, is essential for disease control.

The sequence similarity observed for samples originating from the three countries forming part of the second clade, Clade B, indicates that cross-border spread occurs between Mozambique and the two neighbouring countries of South Africa and Zimbabwe (Figure 2). The movement of animals between these three countries could potentially have widespread implications for future disease control interventions in the region (Figure 3). Any disease intervention programmes initiated in these countries will most likely have to rely on collaborative efforts involving all three of the neighbouring countries in order to be successful.

Clade C demonstrates the value of a functioning surveillance network. Prior to this study, no epidemiological information was available for rabies in the north of Mozambique. However, with the addition of a small number of samples from the region, it has become evident that rabies is present in this region. This Clade has brought to light the presence of cross-border spread between Malawi, eastern Zimbabwe and northern Mozambique, despite large gaps in surveillance. This emphasises the need for improved surveillance as well as the decentralisation of diagnosis. The molecular epidemiology has also highlighted the Nampula province as a potential rabies hotspot, where all of the various known genetic cycles of rabies occur (Clades A-C) (Figure 3).

It is important to note that the large empty geographical spaces separating the isolates clustering in Clade C (Figures 2 and 3) do not imply that the Niassa, Zambezia and Tete provinces are rabies free, but rather that very limited disease surveillance is taking place in the north of Mozambique. With the high sequence similarity observed between the samples from the north of Mozambique and the samples originating from Zimbabwe (Mashonaland Central province) and Malawi, it is apparent that numerous instances of cross-border spread may become evident once surveillance is improved. Finally, canine rabies is a relatively new introduction to south-east Africa (Nel & Rupprecht 2007). The movement of people and their pets or work animals between the major cities in south-eastern Africa would explain the genetic homogeneity found among the viruses studied here, despite the long geographical distances and barriers that would impede the spread of rabies.

The only laboratory capable of rabies diagnosis (DFA test) in Mozambique is situated in the capital of Maputo (Maputo province), which is located at the southern tip of the country. Poor roads and the long distance samples have to travel from the north of the country to the CVL could explain why so few samples originate from the northern provinces of the country. To improve sample submission in a cost-effective way, the decentralisation of rabies diagnosis would decrease the distance samples have to be transported, whilst theoretically increasing the number of samples submitted. Because of the low capital investment requirements and comparable diagnostic sensitivity, the DRIT would be the ideal test for decentralised implementation and could serve to provide a more accurate representation of the rabies burden in the surrounding area. Through the use of the DRIT, surveillance can be improved in areas where it has typically been lacking.

It has been demonstrated on various occasions that the only feasible way to eliminate human rabies is to control and eliminate the disease from canine populations. The strategic vaccination of dog populations should be directed by effective, accurate and cost-effective surveillance networks, as illustrated in this study through the use of the DRIT. Although the establishment of a foundational surveillance system is of utmost importance, it is clear that basic epidemiological information is not sufficient if a higher epidemiological resolution is needed. Basic burden data coupled with molecular epidemiology can improve the resolution of surveillance by determining focal points for rabies intervention.

## Conclusion

In support of previous observations, this study further demonstrates the cross-border spread of rabies between neighbouring countries in Africa, thus impacting disease control and elimination efforts for the region as a whole. Elimination of canine rabies is possible and should be the ultimate objective of rabies control initiatives. To achieve this, regional and continental strategies that rely on and encourage effective national programmes are needed. Without the cooperation of neighbouring countries, rabies will continue to encroach on rabies-free areas or areas where disease elimination is underway.

The results of this study do not reflect negatively on the competency of the laboratory (CVL), but rather highlight the technical challenges faced in developing countries. These infrastructural challenges can affect the condition of samples during or after shipment and storage. Considering time and cost-effectiveness, accuracy and ease of use, the DRIT continues to demonstrate its potential value as a key player in the quest to improve rabies surveillance in the developing world.

## References

[CIT0001] Adawa D.A.Y., Abdullahi S.U., Ogunkoya A.B. & Rupprecht C.E, 2014, ‘Efficacy of a direct rapid immunohistochemical test (DRIT) for rabies detection in Nigeria’, *African Journal For Biomedical Research* 17, 101–107.

[CIT0002] Aguiar T.D., Teixeira M.F., Costa E.C., Vitaliano A.B., Teles C.H., Barroso I.C. et al., 2013, ‘Medium-term cryopreservation of rabies virus samples’, *Revista da Sociedade Brasileira de Medicina Tropical* 46(6), 678–683. 10.1590/0037-8682-0135-2013 24474007

[CIT0003] Ali A., Sifer D., Getahun G. & Aklilu M, 2014, ‘Comparison of direct, rapid immunohistochemical test performance with direct fluorescent antibody test for rabies diagnosis in Ethiopia’, *Open Journal of Biochemistry* 1(1), 49–55. https://doi.org/10.15764/BIOC.2014.01006

[CIT0004] Bishop G.C., Durrheim D.N., Kloeck P.E., Godlonton J.D., Bingham J., Speare R. et al., 2010, *Rabies: Guide for the medical, veterinary and allied professions*, 2nd edn, Blumberg L., Weyer J., Pienaar H., Markotter W. & Rabies Advisory Group (eds.), pp. 1–82, Department of Agricultue and Department of Health, Pretoria.

[CIT0005] CDC, 2003, *Protocol for postmortem diagnosis of rabies in animals by direct fluorescent antibody testing*, viewed 27 September 2016, from http://www.cdc.gov/rabies/pdf/rabiesdfaspv2.pdf

[CIT0006] Coertse J., Weyer J., Nel L.H. & Markotter W, 2010, ‘Improved PCR methods for detection of African rabies and rabies-related lyssaviruses’, *Journal of Clinical Microbiology* 48(11), 3949–3955. 10.1128/JCM.01256-10 20810772 PMC3020803

[CIT0007] Coetzer A, 2013, ‘Comparison of biotinylated monoclonal and polyclonal antibodies in an evaluation of a direct rapid immunohistochemical test for the routine diagnosis of rabies in southern Africa’, MSc thesis, Department of Microbiology and Plant Pathology, University of Pretoria. 10.1371/journal.pntd.0003189 PMC4177867 25254652

[CIT0008] Coetzer A., Nel L.H. & Rupprecht C.E, 2014a, ‘Demonstration of *Lyssavirus* antigens by a direct rapid immunohistochemical test’, in Rupprecht C.E. & Nagarajan J. (eds.), *Current laboratory techniques in rabies diagnosis, research and prevention*, 1st edn, pp. 27–36, Elsevier Ltd, San Diego, CA.

[CIT0009] Coetzer A., Sabeta C.T., Markotter W., Rupprecht C.E. & Nel L.H, 2014b, ‘Comparison of biotinylated monoclonal and polyclonal antibodies in an evaluation of a direct rapid immunohistochemical test for the routine diagnosis of rabies in southern Africa’, *PLoS Neglected Tropical Diseases* 8(9), e3189 10.1371/journal.pntd.0003189 25254652 PMC4177867

[CIT0010] Cohen C., Sartorius S., Sabeta C.T., Zulu G., Paweska J., Mogoswane M. et al., 2007, ‘Epidemiology and molecular virus characterization of reemerging rabies, South Africa’, *Emerging Infectious Diseases* 13(12), 1879–1886. 10.3201/eid1312.070836 18258039 PMC2874428

[CIT0011] Dean D.J., Abelseth M.K. & Atanasiu P, 1996, ‘The fluorescent antibody test’, in Meslin F.-X., Kaplan M.M. & Koprowski H. (eds.), *Laboratory techniques in rabies*, 4th edn, pp. 88–89, World Health Organization, Geneva.

[CIT0012] Dürr S., Naïssengar S., Mindekem R., Diguimbye C., Niezgoda M., Kuzmin I. et al., 2008, ‘Rabies diagnosis for developing countries’, *PLoS Neglected Tropical Diseases* 2(3), e0000206 10.1371/journal.pntd.0000206 PMC2268742 18365035

[CIT0013] Hall T.A, 1999, ‘BioEdit: A user-friendly biological sequence alignment editor and analysis program for Windows 95/98/NT’, *Nucleic Acid Symposium* 41, 95–98.

[CIT0014] Hampson K., Coudeville L., Lembo T., Sambo M., Kieffer A., Attlan M. et al., 2015, ‘Estimating the global burden of endemic canine rabies’, *PLoS Neglected Tropical Diseases* 9(4), e0003709 10.1371/journal.pntd.0003709 25881058 PMC4400070

[CIT0015] International Coordinating Group (ICG), 2014, Report of the sixth meeting of the International Coordinating Group of the World Health Organization and the Bill & Melinda Gates Foundation project on eliminating human and dog rabies, Durban, South Africa, 22–24 September.

[CIT0016] Lembo T., Niezgoda M., Velasco-Villa A., Cleaveland S., Ernest E. & Rupprecht C.E, 2006, ‘Evaluation of a direct, rapid immunohistochemical test for rabies diagnosis’, *Emerging Infectious Diseases* 12(2), 310–313. 10.3201/eid1202.050812 16494761 PMC3294322

[CIT0017] Lopes M.C., Venditti L.L.R. & Queiroz L.H, 2010, ‘Comparison between RT-PCR and the mouse inoculation test for detection of rabies virus in samples kept for long periods under different conditions’, *Journal of Virological Methods* 164(1–2), 19–23. 10.1016/j.jviromet.2009.11.017 19931312

[CIT0018] Madhusudana S.N., Subha S., Thankappan U. & Ashwin Y.B, 2012, ‘Evaluation of a direct rapid immunohistochemical test (dRIT) for rapid diagnosis of rabies in animals and humans’, *Virologica Sinica* 27(5), 299–302. 10.1007/s12250-012-3265-6 23055005 PMC8218131

[CIT0019] Mani R.S. & Madhusudana S.N, 2013, ‘Laboratory diagnosis of human rabies: Recent advances’, *Scientific World Journal* 2013, 569712 10.1155/2013/569712 24348170 PMC3848253

[CIT0020] Mansvelt P.R, 1962, ‘Rabies in South Africa. Field control of the disease’, *Journal of the South African Veterinary Association* 33, 313–319.

[CIT0021] McElhinney L.M., Fooks A.R. & Radford A.D, 2008, ‘Diagnostic tools for the detection of rabies virus’, *European Journal of Companion Animal Practice* 18(3), 224–231.

[CIT0022] McElhinney L.M., Marston D.A., Brookes S.M. & Fooks A.R, 2014, ‘Effects of carcase decomposition on rabies virus infectivity and detection’, *Journal of Virological Methods* 207, 110–113. 10.1016/j.jviromet.2014.06.024 25010791

[CIT0023] Mkhize G.C., Ngoepe E.C., D Plessis B.J.A., Reininghaus B. & Sabeta C.T, 2010, ‘Re-emergence of dog rabies in Mpumalanga province, South Africa’, *Vector Borne and Zoonotic Diseases* 10(9), 921–926. 10.1089/vbz.2009.0109 20370435

[CIT0024] Nel L.H, 2013, ‘Discrepancies in data reporting for rabies, Africa’, *Emerging Infectious Diseases* 19(4), 529–533. 10.3201/eid1904.120185 23628197 PMC3647406

[CIT0025] Nel L.H. & Markotter W, 2007, ‘Lyssaviruses’, *Critical Reviews in Microbiology* 33(4), 301–324. 10.1080/10408410701647602 18033596

[CIT0026] Nel L.H. & Rupprecht C.E, 2007, ‘Emergence of lyssaviruses in the old world: The case of Africa’, *Current Topics in Microbiology and Immunology* 315(1), 161–193. 10.1007/978-3-540-70962-6_8 17848065

[CIT0027] Perrin P, 1973, ‘Techniques for the preparation of rabies conjugates’, in Meslin F.-X., Kaplan M.M. & Kaprowski H. (eds.), *Laboratory techniques in rabies*, 4th edn, pp. 433–441, World Health Organization, Geneva.

[CIT0028] Rodrigues F, 2011, Southern and Eastern Africa Rabies Group, in *Rabies in Mozambique: Update*, Maputo, Mozambique.

[CIT0029] Rojas Anaya E., Loza-Rubio E., Banda Ruiz V.M. & Hernández Baumgarten E, 2006, ‘Use of reverse transcription-polymerase chain reaction to determine the stability of rabies virus genome in brains kept at room temperature’, *Journal of Veterinary Diagnostic Investigation* 18(1), 98–101. 10.1177/104063870601800115 16566265

[CIT0030] Russel C.A., Real L.A. & Smith D.L, 2006, ‘Spatial control of rabies on heterogeneous landscapes’, *PloS One* 1(1), e27 10.1371/journal.pone.0000027.g001 17183654 PMC1762310

[CIT0031] Sabeta C.T., Mansfield K.L., McElhinney L.M., Fooks A.R. & Nel L.H, 2007, ‘Molecular epidemiology of rabies in bat-eared foxes (*Otocyon megalotis*) in South Africa’, *Virus Research* 129, 1–10. 10.1016/j.virusres.2007.04.024 17537536

[CIT0032] Sacramento D., Bourhy H. & Tordo N, 1991, ‘PCR technique as an alternative method for diagnosis and molecular epidemiology of rabies virus’, *Molecular and Cellular Probes* 5, 229–240. 10.1016/0890-8508(91)90045-L 1714538

[CIT0033] Saturday G.A., King R. & Fuhrmann L, 2009, ‘Validation and operational application of a rapid method for rabies antigen detection’, *U.S. Army Medical Department Journal January - March*, 42–45. 20088045

[CIT0034] Southern and Eastern African Rabies Group, n.d., *Epidemiology of rabies*, viewed 28 January 2015, from http://searg.info/doku.php?id=aboutrabies:rabiesepidemiology

[CIT0035] Swanepoel R., Barnard B.J., Meredith C.D., Bishop G.C., Brückner G.K. & Foggin C.M, 1993, ‘Rabies in southern Africa’, *Onderstepoort Journal of Veterinary Research* 60, 325–346. 7777317

[CIT0036] Townsend S.E., Lembo T., Cleaveland S., Meslin F.X., Miranda M.E., Putra A.A.G. et al., 2013, ‘Surveillance guidelines for disease elimination: A case study of canine rabies’, *Comparative Immunology, Microbiology and Infectious Diseases* 36(3), 249–261. 10.1016/j.cimid.2012.10.008 PMC3693035 23260376

[CIT0037] Von Teichman B.F., Thomson G.R., Meredith C.D. & Nel L.H, 1995, ‘Molecular epidemiology of rabies virus in South Africa: Evidence for two distinct virus groups’, *Journal of General Virology* 76(1), 73–82. 10.1099/0022-1317-76-1-73 7844544

[CIT0038] Wacharapluesadee S. & Hemachudha T, 2010, ‘Ante- and post-mortem diagnosis of rabies using nucleic acid-amplification tests’, *Expert Review of Molecular Diagnostics* 10(2), 207–218. 10.1586/erm.09.85 20214539

[CIT0039] Weyer J. & Blumberg L, 2007, ‘Rabies: Challenge of diagnosis in resource poor countries’, *Infectious Diseases Journal of Pakistan*, Brief Comm, 86–88.

[CIT0040] World Health Organization (WHO), 2013, *WHO expert consultation on rabies*, Second report, WHO Technical Report Series 982, pp. 1–141, Geneva, Switzerland.

[CIT0041] Zulu G.C., Sabeta C.T. & Nel L.H, 2009, ‘Molecular epidemiology of rabies: Focus on domestic dogs (*Canis familiaris*) and black-backed jackals (*Canis mesomelas*) from northern South Africa’, *Virus Research* 140(1–2), 71–78. 10.1016/j.virusres.2008.11.004 19061924

